# A Multi-Modal Under-Sensorized Wearable System for Optimal Kinematic and Muscular Tracking of Human Upper Limb Motion

**DOI:** 10.3390/s23073716

**Published:** 2023-04-03

**Authors:** Paolo Bonifati, Marco Baracca, Mariangela Menolotto, Giuseppe Averta, Matteo Bianchi

**Affiliations:** 1 Research Center “E. Piaggio”, Department of Information Engineering, University of Pisa, Largo Lucio Lazzarino 1, 56126 Pisa, Italy; 2 Department of Control and Computer Engineering, Politecnico di Torino, 10129 Torino, Italy

**Keywords:** human multimodal motion tracking, optimal design, Sensor Fusion, IMUs, sEMG sensors, upper limb, wearable sensing

## Abstract

Wearable sensing solutions have emerged as a promising paradigm for monitoring human musculoskeletal state in an unobtrusive way. To increase the deployability of these systems, considerations related to cost reduction and enhanced form factor and wearability tend to discourage the number of sensors in use. In our previous work, we provided a theoretical solution to the problem of jointly reconstructing the entire muscular-kinematic state of the upper limb, when only a limited amount of optimally retrieved sensory data are available. However, the effective implementation of these methods in a physical, under-sensorized wearable has never been attempted before. In this work, we propose to bridge this gap by presenting an under-sensorized system based on inertial measurement units (IMUs) and surface electromyography (sEMG) electrodes for the reconstruction of the upper limb musculoskeletal state, focusing on the minimization of the sensors’ number. We found that, relying on two IMUs only and eight sEMG sensors, we can conjointly reconstruct all 17 degrees of freedom (five joints, twelve muscles) of the upper limb musculoskeletal state, yielding a median normalized RMS error of 8.5% on the non-measured joints and 2.5% on the non-measured muscles.

## 1. Introduction

The evaluation of the musculoskeletal state of the human body is crucial for different applications, such as rehabilitation and assistive technologies [1], sportsmen monitoring [2,3] and human-robot interaction and collaboration [4]. Such a monitoring is also important to prevent possible work-related musculoskeletal disorders, providing tools for a proper ergonomics evaluation [5,6,7] informed by suitably devised bio-mechanical models [8].

Considering the degrees of freedom (DoFs) of the human body, i.e., joints and muscular sites, a correct tracking of human kinematics and muscular activity would require the acquisition of a large amount of data and the usage of many sensors [9]. To record muscle activation, the standard solution is surface electromyography (sEMG), which relies on the usage of electrodes fastened on the skin that measure the electric signal (expressed in mV) produced by muscles. For kinematic measures, instead, the gold standard has been traditionally provided by optical systems, which can monitor human body motion by recording the 3D position in the time of active or passive optical markers. These systems have been proved to be efficient and reliable, but they come with limitations of the operating space. Furthermore, occlusions can also occur, thus affecting the overall reconstruction performance. This problem also affects other marker-less, camera-based methods that have been proposed [10]. A solution to address the problem of environmental occlusion was presented in [11], where the authors exploited radio signals to estimate human pose through walls. However, this approach cannot be generalized to any distance from the sensor, or any type of occlusion, e.g., induced by the presence of other people.

Wearable solutions have emerged as a promising paradigm to enable ecological monitoring, overcoming the workspace limits that affect camera-based methods. Ergonomics and form-factor related considerations tend to discourage the usage of cumbersome sensors. Under this regard, inertial measurement unit (IMU)-based approaches have found fertile ground for kinematic tracking, thanks to their compact design and reduced costs [12,13].

However, to obtain a full biomechanical assessment of the human body, kinematic information is not sufficient but it should be complemented with the recording of muscular activation, e.g., to correctly evaluate the fatigue level of the user during task execution [14,15,16]. Simultaneous acquisition and fusion of muscular and kinematic information have been proposed, e.g., in [17], where measurements from IMUs and mechanomyography were exploited for classifying different actions of the lower limb and for evaluating pathological state. Of note, wearable solutions (eventually complemented, in some cases, by cost considerations) tend to discourage the usage of many sensors mounted on the body, which could negatively impact the form factor and the wearability of the device [18]. A possible approach to tackle this issue is to exploit the covariation schemes between functional elements or DoFs of our body, usually named as *motor synergies* [19]. Indeed, several works demonstrated the existence of correlation patterns between different joints and/or muscles in the upper [20,21,22,23] and lower limb [24,25]. The underlying idea is that the actuation of a large number of DoFs can be described as a linear combination of a smaller number of generators. In terms of actuation schemes, this concept has been profitably exploited in robotics for the design [26], planning [27,28] and control [29] of anthropomorphic devices, with a special focus on robotic hands and manipulators. In all these cases, a small number of independent actuation variables can be combined to drive a larger number of DoFs in a human-like fashion.

Interestingly, the same paradigm can also be used to inform simplified sensing strategies of human motion. In [30], we demonstrated that it is possible to complement scarce and noisy sensory information on hand grasping posture by fusing it with a priori data through minimum variance estimation (MVE). A priori data represented the most frequent human grasping postures organized in terms of interjoint covariation patterns. In [31], we further built on this approach and identified which were the optimal hand joints that yield the minimization in average of the reconstruction error, exploiting the minimization of the a posteriori covariance matrix. These results allowed us to design a wearable sensing glove to reconstruct the hand pose, relying on a lower number of sensors [32]. However, these approaches are based on the assumption that the a priori information is related to static postures, and their application to the estimation of temporal trajectories cannot be performed in a straightforward manner. Additionally, it is hard to develop a trustworthy estimation of the covariance matrix from heterogeneous data due to the concurrent reconstruction of multimodal motion-related data (such as joint angles and EMG signals) [33]. In [34], we proposed to generalize these methods for the estimation of multi-modal time-varying data of the upper limb. The method built upon the existence of covariation patterns in human upper limb motions, as we demonstrated in [23] and the usage of functional analysis for reconstructing the whole trajectory over time and estimating the covariance matrix. In brief, a base of functional Principal Components (fPCs), derived in advance from a collection of upper limb joint motion profiles of daily living activities, was employed to map the temporal measurements of a reduced number of joints and muscles on the extended state space of weights and average trajectories/muscles envelopes. The state missing part was then reconstructed using MVE. The temporal evolution of the entire muscle-skeletal system is then appropriately integrated with the estimated extended state.

However, in [34], the analysis was performed assuming as state variables the joint angular values and the muscle envelopes, while the non-linear mapping between sensors and state variables was not considered. In this paper, we build upon our previous work and extend the method to design an under-sensorized wearable system for multimodal acquisition of human upper limb trajectories. We assume to have at disposal IMUs for kinematic recording and surface sEMGs for muscular activity acquisition, and that their number is not in a bijective relation with all the DoFs used to describe the whole muscle-skeletal status. We generalize the optimal sensing setup identified in [34] to the more challenging case in which one sensor may record the activity of multiple DoFs. Indeed, since the goal is now to reduce the number of employed sensor elements, instead of selecting the single optimal degrees of freedom, i.e., the ones that are associated with a reduced estimation uncertainty, our targeted optimal joint angles are those that enable a compromise between optimal reconstruction and the minimization of the sensing resource in use. To target both objectives, we select as measures the shoulder joints. In this way, we minimize the differences with respect to the optimal setup reported in [34]. Finally, we build a real prototype of an optimal under-sensorized setup for upper limbs (i.e., which has a number of elements lower than the number required to measure all states of the system), with only two IMUs to retrieve angles from the shoulder by implementing an Unscented Kalman Filter (UKF). We integrated these measurements with the optimal sEMGs identified in [34], discarding the other ones, and using a commercially available fully sensorized solution (i.e., Xsens) to have a ground truth for result comparison. Extensive tests on a dataset collected with our framework demonstrate that our method can effectively compensate for missing recordings (corresponding to two out of five joint angles and four out of twelve sEMG signals), with minimum impact on the estimation error, achieving a median normalized RMS error of 8.5% on the non-measured joints and of 2.5% on the non-measured EMGs.

The paper is organized as follows: we first summarize the theory underpinning our optimization method and its application to our case, with the UKF implementation for retrieving shoulder angles; then, we discuss the experimental setup for data acquisition and system testing, and the results.

## 2. Methods

### 2.1. Theoretical Foundations: Minimum Variance Estimation (MVE)

**Figure 1:**
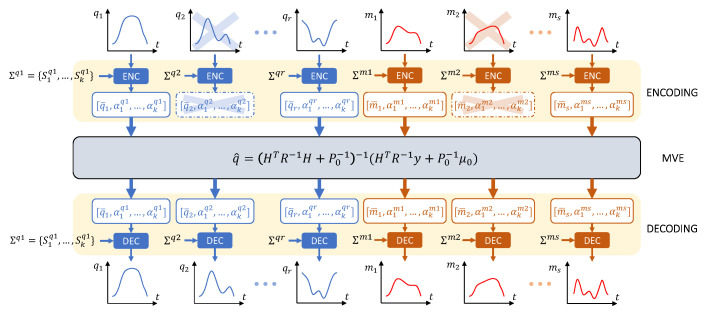
Schematic flow of the estimation procedure. First temporal signals are mapped on the weight vector through the fPC bases (*Encoding*). After that, *Minimum Variance Estimation* (MVE) fuses the encoded measures with *a priori* knowledge to estimate for the missing part of measures. In the end, the estimated weight vector is converted back to the temporal domain (*Decoding*).

Here we briefly summarize the results in [34]. The idea is to translate the recorded movements into a static representation, use it to obtain the *a priori* covariance matrix, perform the estimation and then re-express the movements in the temporal domain. To do this, we define three separate phases in this method: encoding, estimation and decoding. The procedure is briefly depicted in Figure 1.

#### 2.1.1. Encoding and Decoding Phases: Functional Principal Component Analysis

Functional Principal Component Analysis (fPCA) is a statistical method to identify functional primitives from time-varying data. In this section, we will provide a brief introduction to the theory, while werefer to [35] for more details. For the sake of simplicity, since each DoF can be analyzed separately from the others with this method, the equations will be defined for a single joint. Let us consider *N* independent observations of joint temporal evolution $q_{1}\left(t\right),\ldots ,q_{N}\left(t\right)$ with t∈[0,1]. A generic motion can be decomposed as a weighted sum of basis elements $S_{i}\left(t\right)$, known as *functional Principal Components* (fPCs):

(1) $$q\left(t\right)≃\bar{q}+S_{0}\left(t\right)+\sum_{i=1}^{s_{max}}\alpha_{i}S_{i}\left(t\right)$$

where $\bar{q}$ is the average value of the joint, $S_{0}\left(t\right)$ is the average trajectory across all the trajectories in the dataset, $\alpha_{i}$ is the weight associated with the $i^{th}$ basis element $S_{i}\left(t\right)$ and $s_{max}$ is the number of basis elements. The output of fPCA is a basis of functions $\left\{S_{1}\left(t\right),\ldots ,S_{s_{max}}\left(t\right)\right\}$ which maximizes the explained variances of joint motions throughout the whole dataset. For more detail on how these fPCs can be extracted, we refer the interested reader to [35].

This decomposition can be done for each DoF of the considered system, regardless of whether it is a kinematic or muscular measure, and it allows us to translate the trajectories from the time domain to the fPCs weight domain. Then, it is possible to represent movments that an extended state $x_{e}$, which does not depend on time, to represent movements. Given *M* degrees of freedom and using *k* fPCs for the decomposition, the extended state, from which we can compute the covariance matrix $P_{0}$, can be defined as:

(2) $$x_{e}={\left[\begin{array}{c} {\bar{x}}_{1}\;\alpha_{1}^{x_{1}}\;\ldots \;\alpha_{k}^{x_{1}}\;\left|\;\ldots \;\right|\;{\bar{x}}_{M}\;\alpha_{1}^{x_{M}}\;\ldots \;\alpha_{k}^{x_{M}} \end{array}\right]}^{T}$$

where $x_{i}$ is the generic *i-th* degree of freedom. This new state definition is the output of the encoding phase and it will be used as the state of the MVE.

When performing fPCA to decompose a signal, the noise is usually represented by the higher-order components. Indeed, the fPC decomposition allows truncating this basis to include only a few elements ordered based on the variance they can account for, giving an additional tool to minimize the effect of noise in the a priori covariance matrix, which will be introduced in the next section. In our work, we used the first 7 functional Principal Components out of 10, which can account for a cumulative variance greater than 95% for each DoF.

Regarding the decoding phase, given the estimation of the extended state ${\hat{x}}_{e}$ provided by the MVE, we can return to the temporal domain by combining the fPCs through (1).

#### 2.1.2. Estimation Phase: Minimum Variance Estimation

The Minimum Variance Estimation (MVE) approach is an algorithm that leverages on the information of a set of *a priori* observations, organized in terms of mean $\mu_{0}$ and covariance matrix $P_{0}$, to estimate missing or noisy measurements. In the following, we will briefly describe this method, while we address to [30] for more details.

Considering a vector of measures $y\in R^{d}$ provided by a selection of *d* sensors, and assuming a linear relationship between the state variables $x\in R^{l}$ and the measures *y*, then y=Hx+ν, where $H\in R^{d,l}$ is a full row rank measurement matrix and ν is the measurement noise. The goal is to estimate *x* given *y* when d<l. If the number of realizations of *x* (collected in a matrix of *a priori* $X\in R^{l,N}$) is large enough, the covariance matrix results:

(3) $$P_{0}=\frac{\left(X-\bar{x}\right){\left(X-\bar{x}\right)}^{T}}{N-1}$$

where $\bar{x}$ is a matrix whose columns contain the average $\mu_{0}$ of *X*. Given $P_{0}$, the best estimate $\hat{x}$ of *x* is the vector that solves the following optimization problem:

(4) $$\hat{x}=\mathrm{argmin}\frac{1}{2}{\left(x-\mu_{0}\right)}^{T}P_{0}^{-1}\left(x-\mu_{0}\right).$$

Assuming that ν is the zero mean Gaussian noise with covariance matrix *R*, the solution of (4) can be found in a closed form as:

(5) $$\hat{x}={\left(H^{T}R^{-1}H+P_{0}^{-1}\right)}^{-1}\left(H^{T}R^{-1}y+P_{0}^{-1}\mu_{0}\right).$$

We can also define the *a posteriori* covariance matrix, which contains the information regarding the uncertainty of the associated state estimation, as:

(6) $$P_{P}={\left(H^{T}R^{-1}H+P_{0}^{-1}\right)}^{-1}$$

Its maximum eigenvalue is a measure of the estimation uncertainty and its dependence on the selection matrix *H* allows us to link the quality of the estimation with the sensor placement. Hence, we can set up the following optimization problem to search for the best selection matrix $H_{opt}$ given a certain number of sensors:

(7) $$H_{opt}=\underset{H}{\mathrm{argmin}}\;\sigma_{max}\left(P_{P}\left(H\right)\right)$$

There are different ways to solve this optimization. However, in our case, we have to preserve the particular structure of the selection matrix. Indeed, the matrix H is composed by squared blocks $H_{i}$ of dimension k+1, each of which is a diagonal matrix corresponding to the average signal and the first *k* fPC coefficients of the *i-th* degree of freedom, which represent the extended state in (2). To deal with this constraint, in our previous work [34], we used a genetic algorithm.

### 2.2. Musculoskeletal Model and Sensor Choice

**Figure 2:**
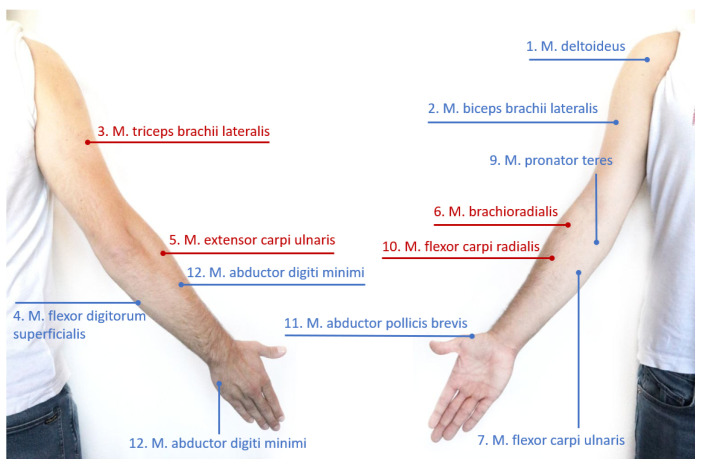
EMG sensor placement in accordance with SENIAM recommendations (back and front views of the right arm). In blue, the muscles used as measures in the MVE algorithm; in red, the estimated muscles.

**Figure 3:**
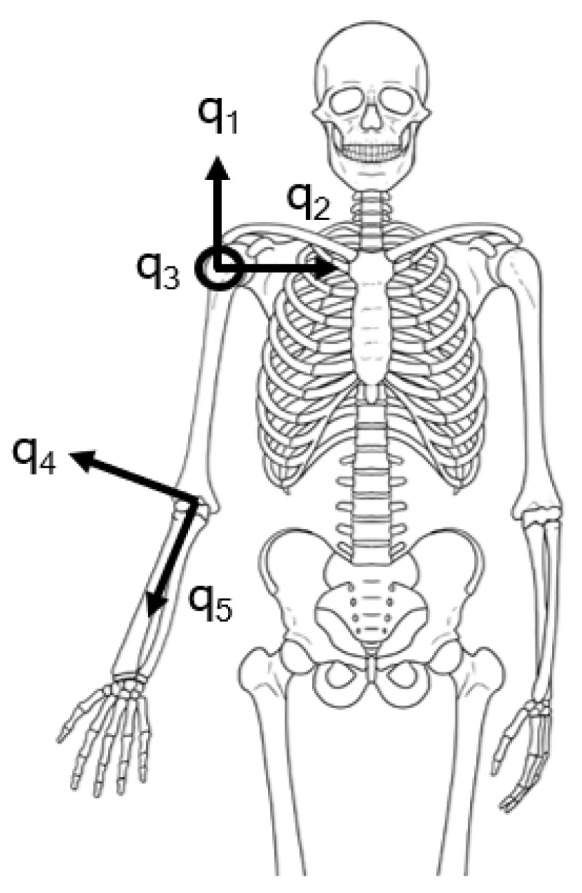
Kinematic model of the human arm (the angle $q_{3}$ is directed outwards).

We considered the same arm muscles (shown in Figure 2) and the same kinematic model (represented in Figure 3) composed of three rotational joint for the shoulder and two for the elbow reported in [34].

In [34] the authors demonstrated that a good estimation of the biomechanical state of the arm can be reached measuring 3 joint angles ($q_{1}$, $q_{3}$, $q_{4}$ in Figure 3) and 8 muscular activation signals (indices 1, 2, 4, 7, 8, 9, 11, 12 in Figure 2). While the muscles optimal selection can be easily translated in the optimal sEMG sensor placement, for the kinematic measurements this is not necessarily true, since IMUs can capture the motion of several DoFs, depending on their placement. Indeed, usually two IMUs are placed before and after the anatomical articulation to estimate the joint angles of the kinematic model. To implement the results obtained in [34], a minimum number of 3 IMUs (one on the shoulder, one on the arm and one on the forearm) would be required. Since we are not assuming to measure every single joint independently from each other, moving from a discrete optimization to a continuous one, our goal is now to reduce the number of sensor elements while maximizing the lowest eigenvalue of the *a posteriori* covariance matrix $P_{p}$. Therefore, the idea is to select a sub-optimal set of joint angles (i.e., the ones of the shoulder $q_{1}$, $q_{2}$, $q_{3}$), which differs from the optimal case for just one DoF, but it requires only two IMUs for sensing.

### 2.3. Unscented Kalman Filter for Joint Angles Estimation via IMUs

Since the kinematic state of the upper-limb, and in particular the joint angles *q* and joint angular velocities $\dot{q}$, cannot be directly measured, a possible solution is based on an Unscented Kalman Filter (UKF) [36], which fuses the information given by a kinematic model of the arm with the measures of gyroscopes and accelerometers collected by two IMUs. Furthermore, the integration of magnetic field measures allows us to avoid the drifting behavior of the inertial sensors, which drastically limits the performance of the estimator.

Since we are solely interested in the measurement of the shoulder angles, from now on we can define the shoulder joint vector as $q={\left[\begin{array}{c} q_{1},\;\;q_{2},\;\;q_{3} \end{array}\right]}^{T}$. The state space model of our UKF is based on the state $x\left(k\right)={\left[\begin{array}{c} q\left(k\right),\;\dot{q}\left(k\right) \end{array}\right]}^{T}$, which contains the shoulder joints angles and the respective joint angular velocities at time *k*. The dynamic model of the *i*-th joint angle can be described with a first-order approximation as:

(8) $$\left\{\begin{array}{c} q_{i}\left(k+1\right)=q_{i}\left(k\right)+{\dot{q}}_{i}\left(k\right)\cdot \Delta T+w_{q}\left(k\right)\\ {\dot{q}}_{i}\left(k+1\right)={\dot{q}}_{i}\left(k\right)+w_{\dot{q}}\left(k\right) \end{array}\right.$$

where ΔT is the sampling time and the state is modelled as a random walk with Gaussian white noises $w_{q}$ and $w_{\dot{q}}$.

The definition of the measurement model is based on the relationship between the inertial and magnetic field variables ω, *a* and *m* in the frames attached to the scapula IMU $\left\{S_{R}\right\}$ and the arm IMU $\left\{A_{R}\right\}$, passing through each pair of consecutive Denavit-Hartenberg frames {i} and {i+1}. Assuming that the only value measured by the accelerometers is the gravitational acceleration (i.e., the linear acceleration of the IMU and the Coriolis and centripetal accelerations are negligible) and that the two magnetometers are affected by the same disturbances, it is possible to write:

(9) $$\left\{\begin{array}{c} \omega_{i+1}^{i+1}=R_{i+1,i}\;\left(\omega_{i}^{i}+z_{i}\cdot {\dot{\theta }}_{i+1}\right)\\ a_{i+1}^{i+1}=R_{i+1,i}\;a_{i}^{i}\\ m_{i+1}^{i+1}=R_{i+1,i}\;m_{i}^{i} \end{array}\right.$$

where $R_{i+1,i}=R_{i+1,i}\left(q_{i+1}\right)$ and ${\dot{\theta }}_{i+1}={\dot{q}}_{i+1}$ when the relative motion of two consecutive frames depends on a revolute joint $J_{i+1}$ in between, following the Denavit-Hartenberg parametrization (in this case, $z_{i}$ is the i−th joint axis), while $R_{i+1,i}$ is constant and ${\dot{\theta }}_{i+1}=0$ otherwise.

The goal is to write the relationship between the measured variables in the frame $\left\{S_{R}\right\}$ of the scapula IMU and those in the frame $\left\{A_{R}\right\}$ attached to the arm IMU using the state variables. To do this, we first define the generic vector $\xi_{n}={\left[\begin{array}{c} \omega_{n}^{n},\;a_{n}^{n},\;m_{n}^{n} \end{array}\right]}^{T}\in R^{9}$, which contains all the variables associated to the n-*th* IMU in its frame {n}.

Choosing as measures $y=\xi_{A_{R}}$, i.e., the IMU measurements after the processing described in Section 3.1, the measurement model depends only on the state and on the output noise and results in:

(10) $$\left\{\begin{array}{c} h=h(q,\dot{q},\xi_{S_{R}},\nu_{S})\\ y=\xi_{A_{R}}+\nu_{A} \end{array}\right.$$

The computation of *h* for the acceleration and magnetic field components is based on the simple relations $a_{A_{R}}=R_{A_{R},S_{R}}a_{S_{R}}$ and $m_{A_{R}}=R_{A_{R},S_{R}}m_{S_{R}}$, where the transformation $R_{A_{R},S_{R}}$ corresponds to:

(11) $$R_{A_{R},S_{R}}=R_{C_{A}}\cdot R_{q}\left(q_{1},q_{2},q_{3}\right)\cdot R_{C_{S}}$$

where $R_{q}\left(q_{1},q_{2},q_{3}\right)$ is the rotation matrix between the Denavit-Hartenberg (D-H) frames, while $R_{C_{A}}$ and $R_{C_{S}}$ are the calibration rotation matrices obtained through the calibration procedure of Section 3.2. So, the acceleration and magnetic components of *h* depend only on *q* and $\xi_{S_{R}}$. The relation between the angular velocities $\omega_{S_{R}}$ and $\omega_{A_{R}}$ can be obtained by following the procedure in (9) from the first frame to the last one; in this case, the output function also depends on $\dot{q}$.

The magnetometer raw data are calibrated through the procedure described in Section 3.1. However, this step does not remove the disturbances that may affect the magnetic sensors, so we modified our UKF to increase the magnetometer noise to weigh this contribution less if a magnetic disturbance is acting on the sensor itself, as done in [37]. Indeed, if the norm of the magnetic field *m* does not fall within a certain range with respect to the normalized value $m_{norm}=1$, we sensibly increase the noise variance of magnetometer measurements inside the output noise covariance matrix R of the UKF. In other terms, the magnetometer noise components $\sigma_{m}^{2}$ inside the matrix R were chosen as:

(12) $$\sigma_{m}^{2}=f\left(||m||-1\right)+\sigma_{const}^{2},$$

where f(·) is a function that depends linearly (or exponentially) on the difference ||m||−1 through a parameter *k* (in our case, f(||m||−1)=k(||m||−1), with k=10).

Hence, the UKF allows us to estimate the shoulder joint angles *q*, leveraging on the inertial and magnetic field measures of the IMUs.

## 3. Experimental Setup

The goal of this experimental setup is to gather a set of data to validate both the UKF for the measurement of shoulder joint angles and the MVE to estimate the missing measurements for biomechanical assessment of the human arm.

We asked 9 able-bodied subjects (6 male and 3 female, age 28.2±2.7, all right-handed) to perform the 30 tasks of daily living described in the SoftPro protocol [38]. Each of these tasks was repeated three times for a total of 90 movements per subject. Participants did not have any physical limitations that could have affected the experimental outcomes. They gave their informed consent to participate. The procedures were approved by the Committee on Bioethics of the University of Pisa (Review No. 30/2020) in accordance with the Declaration of Helsinki. The pose in between movements consisted in resting the right hand flat on the table. Since these 90 movements were recorded in one shot, they were shuffled before being instructed to the subjects, to obtain an homogeneous dataset, not influenced by muscular fatigue.

**Figure 4:**
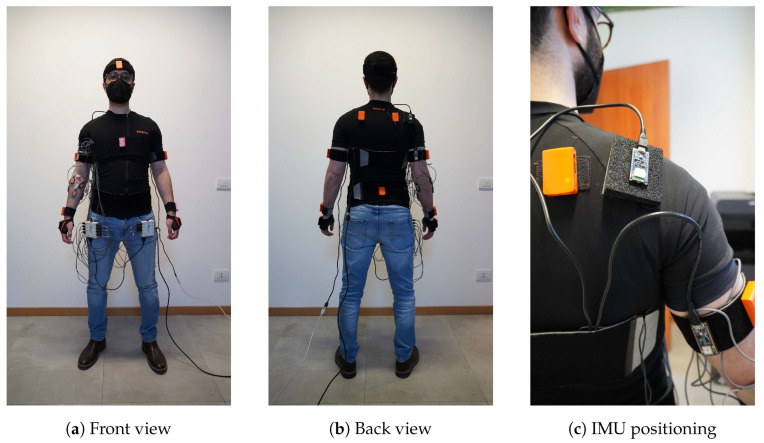
Different views of the complete sensor setup (including the ground truth sensors) used during the experimental phase. The full-body view of the system — composed by the Delsys Bagnoli EMG system (Delsys Inc., Natick, MA, USA), the Xsens MTw Awinda (Movella Inc., Henderson, NV, USA) wearable system and the two LSM9DS1 inertial sensors embedded in Arduino Nano 33 BLE boards (Arduino S.r.l., Monza, Italy) — is shown in (**a**,**b**). A detail of the IMUs positioning is depicted in (**c**).

The kinematic data were recorded with LSM9DS1 inertial sensors embedded in Arduino Nano 33 BLE boards and connected to a computer through serial communication at a sample rate of 120 Hz. The muscular data were recorded with the Delsys Bagnoli EMG System with a sampling frequency of 2400 Hz. The EMG placement followed SE-NIAM guidelines to minimize the cross-talk phenomen between near muscles is the same as the one adopted in the MHH dataset [38]. The EMG signals and the IMU data were recorded through a custom routine which guaranteed the synchronization between them. To validate the Kalman Filter results, we employed as a ground truth the Xsens MTw Awinda wearable system, which returns the upper-body posture of the subject. The kinematic data were recorded at the Xsens maximum sample rate of 60 Hz. To synchronize the Xsens data, collected via proprietary software, with the EMG and IMU signals, we performed Dynamic Time Warping (DTW) [39]. The whole sensor setup is shown in Figure 4.

### 3.1. IMU Processing

Before using the IMU data, it is important to remove constant biases that affect gyroscopes and accelerometers is important. An example of a debiasing routine can be found in [40]. The Arduino Nano 33 BLE boards, which were used for our work, directly provide the acceleration normalized with respect to the gravity acceleration *g* = 9.81 m/s${}^{2}$.

Regarding the magnetic measures, the magnetometer raw data ${}^{B}m_{r}$ in the sensor frame {B} lie on an ellipsoid manifold, as demonstrated in [41]. In the same work, to translate the raw data to the origin of the sensor frame and map them onto the unitary sphere, a Maximum Likelihood Estimator is used to determine the magnetometer optimal calibration parameters: a SE(3) transformation matrix to align the ellipsoid axes with a calibration frame {C} and center it on its origin, and a scaling matrix to stretch the ellipsoid on the unitary sphere. After this mapping, a second step allows us to find the optimal rotation matrix that minimizes the error between the data mapped on the unitary sphere ${}^{C}m$ and the original raw data ${}^{B}m_{r}$.

From a practical point of view, these calibration parameters can be determined with an initial data acquisition, during which the IMU should be rotated in as many configurations as possible. In this way, the shape of the ellipsoid can be better defined, avoiding sampling a small surface of the ellipsoid, for which the measurement noise can badly affect the parameter extraction.

### 3.2. IMU Frames Calibration

Prior to the estimation phase, it is necessary to evaluate the effective orientation of each sensor *X* attached to the body, i.e., to identify the rotation matrices between the sensor frames $\left\{S_{R}\right\}$ and $\left\{A_{R}\right\}$ and the first/last Denavit-Hartenberg frames, respectively.

In this section, we briefly introduce the approach used in our work and we direct the interested reader to [42] for more details. The procedure consists of a two-phase data acquisition: the first part is performed with the subject standing still with the arms straight along the body (N-pose); and in the second part, the subject is asked to slightly bend forward with their arm fixed to their body. These data return two readings of gravity acceleration in two different poses that are used in a series of cross products to define the calibration matrix.

### 3.3. EMG Processing

Surface EMG signals can be affected by different sources of noise (relative motion of soft tissues, bad mechanical or electrical connections, cross-talking between different muscles, etc…). Several works in literature provide solutions to this problem [43,44]. For our application, we took inspiration from [45] and we implemented the following filtering steps: (1) a first order low-pass Butterworth filter with a cutoff frequency of 500 Hz to reduce the high-frequency noise; (2) a first order high-pass Butterworth filter with a cutoff of 20 Hz, which allows us to remove the constant and slowly-changing behaviors; (3) the rectification of the filtered signal; and (4) another first order low-pass Butterworth filter, with a cutoff frequency of 1 Hz, for the extraction of the signal envelope.

### 3.4. From XSENS Quaternions to Joint Angles

For each link *l* of the arm kinematic chain, the XSENS system returns as output the quaternion $Q_{l}$, which expresses the orientation between the frame of the link and the system world frame. So, given the quaternions $Q_{s}$, $Q_{a}$ and $Q_{f}$ of the shoulder, arm and forearm respectively, we estimated the shoulder joint angles $q_{1}$, $q_{2}$ and $q_{3}$ and the elbow angles $q_{4}$ and $q_{5}$ through an Unscented Kalman Filter. Indeed, we can model the dynamics of the i-*th* joint angle as a random walk with Gaussian white noise $w_{q_{i}}$:

(13) $$q_{i}\left(k+1\right)=q_{i}\left(k\right)+w_{q_{i}}$$

Then, we can use as measures $y_{1}$ for the estimation of the shoulder joints the orientation between the shoulder and arm link $y_{1}=Q_{sa}=Q_{s}^{*}⊗Q_{a}$, where ⊗ represents the quaternion product. Similarly, we can express the orientation between the arm and the forearm as $y_{2}=Q_{af}=Q_{a}^{*}⊗Q_{f}$ and use it as the second block of the output vector. So, the related output functions can be described as:

(14) $$\left\{\begin{array}{c} h_{1}=\left[Q_{01}\left(q_{1}\right)⊗Q_{12}\left(q_{2}\right)\right]⊗Q_{23}\left(q_{3}\right)\\ h_{2}=Q_{34}\left(q_{4}\right)⊗Q_{45}\left(q_{5}\right) \end{array}\right.$$

where the generic quaternion $Q_{i,i+1}$ express the orientation between two subsequent Denavit-Hartenberg frames through joint $q_{i}$.

## 4. Results

### 4.1. UKF Validation

To assess the UKF performance, three different metrics were used: the Root Mean Square (RMS) error between joint evolution estimated and the ones of the Xsens, used as ground truth; the Normalized Root Mean Square (NRMS) obtained by normalizing the RMS error with respect to the maximum range reached by each joint and the correlation index between the two signals (the UKF one and the ground truth) to evaluate their similarity in terms of temporal evolution.

Regarding the RMS, we reached a median value of around $10^{\circ }$ (NRMS around 10%), with a performance comparable with other similar solutions presented in the literature [46,47,48], with an RMS error median between 5.2 to $7.9^{\circ }$ in Slade et al., and between 4.95 to $7.03^{\circ }$ in Peppoloni et al. The similarity between the estimated joint trajectories and the reference ones is also high, since it is about 0.93 for all the angles. In Table 1 the detailed results of these three metrics are reported, in terms of median and interquartile range, for each shoulder joint angle.

### 4.2. MVE Validation

**Figure 5:**
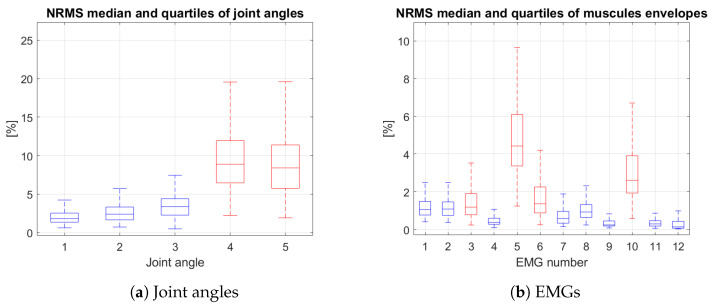
Normalized RMS Error computed for each DoF (measured DoFs in blue, non-measured DoFs in red).

To evaluate the goodness of estimation performed by MVE, we computed the RMS error (RMSE) and NRMS error (NRMSE) comparing it with the ground truth value recorded during tasks execution. In Figure 5 the NRMSE between the real signal and the output of the MVE for each DoF is reported in terms of the median and interquartile range. The measured DoFs are represented in blue, while the estimated ones are in red. For the kinematic part, the NRMS error on the measured joints is about 2.4%. We can notice, as expected, a higher error for the estimated joints with respect to the measured ones, with a median around 8.5%. However, the error level is comparable with the one reached in other solutions presented in the literature [36], with the advantage of a lower number of used sensor elements. For the muscular side, the normalized error level achieved is even lower (maximum median NRMSE just above 4%).

**Figure 6:**
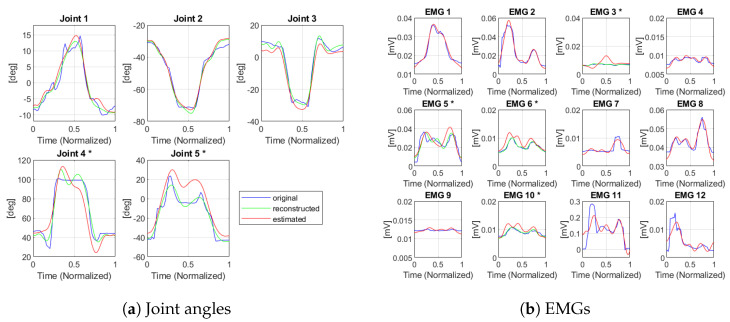
Example of MVE on a movement of the test dataset (in blue: reference movement; in green: movement reconstruction with fPCs; in red: movement obtained through MVE); * = non−measured DoFs.

In terms of RMSE, it reaches 17.1 ± 4.97° for the non-measured joint angles, while for the muscles it is 0.003 ± 0.002 mV (values expressed in median ± interquartile range). This result, compared to the one reported in [34] ($2.18\pm 1.32^{\circ }$ for the joints and 0.003 ± 0.002 mV for the muscles), can be considered sufficiently good, as this joint angle choice was not the optimal one found in [34] and referred to a selection of individual DoFs, but represents an approximation that fulfills the requirement of the minimum number of sensors required for an effective implementation of the measurements. Furthermore, in [34], the kinematic measurements considered for the analysis were provided by a ground truth optical system, while in our case we used the information measured by the IMU-based system we developed - which intrinsically comes with an estimation error, although comparable with or less than the one of the other related works in the literature. An example of a random estimated movement is presented in Figure 6. The not measured DoFs are marked with a star (*). These graphs confirm the results obtained in terms of RMS error.

## 5. Conclusions

The topic of human-robot interaction and collaboration, as well as monitoring the human musculoskeletal state in working environments, has gained increasing attention in recent years. In particular, the assessment of the musculoskeletal state could bring many benefits in terms of improving working conditions and preventing work-related disorders.

In this paper, we present a technological solution that relies on a reduced number of wearable sensing units (IMUs and sEMGs) and provides an estimation of the whole musculoskeletal state.

To do this, we developed an under-sensorized wearable system that exploits the Minimum Variance Estimation approach to assess the bio-mechanical state of the human arm. Additionally, an Unscented Kalman Filter was implemented to directly obtain the joint angle trajectories from the IMUs measurements. This setup was extensively tested through the collection of a new dataset of daily living activities. The obtained results are promising, as they show an average normalized error of 8.5% on the non-measured joints and of 2.5% on the non-measured EMGs. Our system allows an accurate state monitoring, with a reduced number of sensors, thus increasing wearability and reducing discomfort.

Our outcomes can pave the path toward unobtrusive wearable monitoring of multi-modal quantities. First, our theoretical framework allows us to overcome the limitations of data-driven methods that rely on the usage of large training datasets that can be used to complement scarce sensory information. Of note, such a theoretical framework was already presented in our previous publication [34]. Second, we provided, for the first time, an implementation of our optimal design, showing that, with a reduced set of optimally placed sensors, we can reconstruct the whole musculoskeletal state of the upper limb. This under-sensorized implementation leads to the reduction of the number of sensors, enhancing the overall system wearability. While this is already a good achievement for the monitoring of the upper limb, our implementation can pave the path toward whole-body multi-modal sensing, where ergonomics and economic constraints pose even more strict constraints on the number, and quality, of sensors in use.

Starting from these results, the next step will be to compare this approach with a fully data-driven approach (e.g., Deep Generative Adversarial Network [49]) to evaluate the performance of our MVE-based solution with respect to the ones obtained by deep learning techniques, and eventually propose hybrid approaches. Another interesting path to explore would be to find a way to use this setup online, as the functional decomposition requires a movement to be recorded in advance. In the future, we will investigate other techniques for the fusion of IMU and EMG data—and compare and integrate them with our approach also targeting action recognition. It will also be interesting to study zero crossing/time-frequency domain for gesture recognition and HRI [50,51].

Finally, these methods could be extended to the entire human body and therefore assess the entire skeletal and muscular state of a person in different application contexts, such as rehabilitation and human-robot collaboration.

## Figures and Tables

**Table 1:** UKF validation with respect to the Xsens system for shoulder joints estimation. In each column, RMS, Normalized RMS and correlation coefficient are reported in terms of the median and half of the interquartile range.

	RMS Error	NRMS Error	Correlation
$q_{1}$	$10.9\pm 4.6^{\circ }$	11.27±4.72%	0.906±0.084
$q_{2}$	$6.49\pm 1.45^{\circ }$	6.93±1.525%	0.956±0.028
$q_{3}$	$11.1\pm 3.85^{\circ }$	11.01±3.79%	0.930±0.07

## Data Availability

The data are not publicly available due to privacy reasons.
